# Limited evolution of the yellow fever virus 17d in a mouse infection model

**DOI:** 10.1080/22221751.2019.1694394

**Published:** 2019-12-04

**Authors:** Dieudonné Buh Kum, Niraj Mishra, Bram Vrancken, Hendrik Jan Thibaut, Annelies Wilder-Smith, Philippe Lemey, Johan Neyts, Kai Dallmeier

**Affiliations:** a KU Leuven Department of Microbiology, Immunology and Transplantation, Rega Institute, Laboratory of Virology and Chemotherapy, Leuven, Belgium; b Aligos Belgium, Leuven, Belgium; c KU Leuven Department of Microbiology, Immunology and Transplantation, Rega Institute, Laboratory for Clinical and Epidemiological Virology, Leuven, Belgium; d Department of Disease Control, London School of Hygiene and Tropical Medicine, London, United Kingdom; e Heidelberg Institute of Global Health, University of Heidelberg, Heidelberg, Germany

**Keywords:** Yellow fever virus, virus diversity, fractional dosing, YFV-17D, intra-host evolution, live-attenuated vaccine

## Abstract

By infecting mice with the yellow fever virus vaccine strain 17D (YFV-17D; Stamaril®), the dose dependence and evolutionary consequences of neurotropic yellow fever infection was assessed. Highly susceptible AG129 mice were used to allow for a maximal/unlimited expansion of the viral populations. Infected mice uniformly developed neurotropic disease; the virus was isolated from their brains, plaque purified and sequenced. Viral RNA populations were overall rather homogenous [Shannon entropies 0−0.15]. The remaining, yet limited intra-host population diversity (0−11 nucleotide exchanges per genome) appeared to be a consequence of pre-existing clonal heterogeneities (quasispecies) of Stamaril®. In parallel, mice were infected with a molecular clone of YFV-17D which was in vivo launched from a plasmid. Such plasmid-launched YFV-17D had a further reduced and almost clonal evolution. The limited intra-host evolution during unrestricted expansion in a highly susceptible host is relevant for vaccine and drug development against flaviviruses in general. Firstly, a propensity for limited evolution even upon infection with a (very) low inoculum suggests that fractional dosing as implemented in current YF-outbreak control may pose only a limited risk of reversion to pathogenic vaccine-derived virus variants. Secondly, it also largely lowers the chance of antigenic drift and development of resistance to antivirals.

## Introduction

Yellow fever (YF) is an acute haemorrhagic disease caused by the yellow fever virus (YFV), an enveloped, positive-sense RNA virus that belongs to the genus Flavivirus. Other clinically important flaviviruses include the dengue (DENV), Zika (ZIKV), West Nile (WNV), tick-borne encephalitis (TBEV) and Japanese encephalitis (JEV) viruses. Their genome encodes for a single polyprotein that is co- and post-translationally processed into 3 structural proteins and 7 non-structural proteins (NS1-5), the latter responsible for intracellular replication of the viral RNA genome. The open reading frame (ORF) is flanked by untranslated regions (UTR) at the 5’ and 3’ ends, respectively.

YFV infects humans in Africa and South America causing an estimated 84000–170000 severe cases and 29000–60000 deaths per year, with 90% of cases occurring in Africa [1], and the potential threat to global health with introduction into new areas via travellers is greater than ever [2, 3]**.** Accurate data about YF burden are difficult to obtain because of underreporting, limitated passive surveillance, lack of diagnostic capacity in many regions where YF is endemic [4], and occurrence of asymptomatic infection [5, 6]. Given the lack of effective vector control, the most effective strategy to prevent and control yellow fever is by active immunization with high population coverage rates [7]. WHO-prequalified yellow fever vaccines include the yellow fever virus 17D (YFV-17D) prototype vaccine strain, or the YFV-17DD substrain derived thereof. The YFV-17D strain was originally derived from a clinical isolate (Asibi strain) and attenuated by serial passaging in the early 1930s [8, 9]. The live-attenuated YFV-17D is considered as one of the most efficient vaccines ever developed with long immunogenicity [10, 11]. It induces a vigorous, multi-specific and possibly life-long lasting protective immunity in >95% of the vaccinees within 10 days after vaccination [12,13].

YFV-17D has a long-standing historic record and a favourable safety profile [14–16] (about 850 million doses deployed since 1937). All six YF vaccines used today belong to either of the two main sublineages of the original 17D vaccine, 17D-204 and 17DD [17] of which four are prequalified by WHO including the particular 17D-204 vaccine produced in France and licensed under the trade name Stamaril® (Sanofi-Pasteur). However, it also has several drawbacks that hamper its wider use in mass vaccination campaigns, particularly in remote and poorly developed areas: (i) its tedious production in embryonated chicken eggs, (ii) lot-to-lot variabilities posing potential safety concerns of loss of attenuation and reversion to increased virulence, (iii) the inherent thermal instability requiring a cold chain and (iv) the need for needles for parenteral delivery. To circumvent these drawbacks, we developed a plasmid (WO2014174078A1) that allows the convenient direct launching of the YFV-17D viral genome (and that of other RNA viruses) upon injection in the host (WO2014174078A1).

RNA viruses typically replicate to diverse groups of “quasispecies” populations wherein viral diversity may correlate with virulence and pathogenicity, as studied mainly using the poliovirus paradigm [18–22,23]. A major concern for any live-attenuated RNA virus vaccine (such as the YFV-17D) is the emergence of virus variants that lose their attenuation and revert to high virulence. YFV-17D has been empirically developed more than 80 years ago, yet is today still the only vaccine platform to control YFV outbreaks [24]. For vaccine production YFV-17D is grown in a hard to control process in embryonated chicken eggs. The entire process is strictly regulated by WHO recommendation criteria and needs to follow a historically established seed-lot system; continuous quality control involves consistency testing to avoid genetic drift, including mandatory testing for neurovirulence and viscerotropic replication potential in non-human primates. Rare but severe adverse effects (SAE) have been reported for YFV-17D, in particular yellow fever vaccine-associated viscerotropic (YEL-AVD) and neurotropic disease (YEL-AND) [14,25]. Though poorly understood, pathogenesis of YEL-AVD is mostly related to a predisposition of the individual vaccinee, such as functional thymus deficiencies or haematological disorders, and likely not to viral factors, in particular not to virus variants emerging from the vaccine virus quasispecies [25,26]. However, for YEL-AND there is good evidence that an increased virulence of some minority variants that emerge after inoculation and start to dominate the replicating vaccine population may be more directly linked to neurovirulence [27]. The vaccine studied here was of the YF17D-204 lineage that had been reported to be very stable and to accumulate only very few mutations during large-scale production [28]. It is further recognized as one of the YFV-17D vaccines with the lowest reported incidence of SAE [26]. The primary goal of the current study is to assess the genetic stability and diversity (mutation rates and patterns) of YFV-17D during its replication in a vertebrate host. To this end, we used mice deficient in IFN α/β and γ receptors (AG129) that are highly susceptible to YFV, including YFV-17D, and allow maximal expansion of the viral population in the absence of a protective immune response. The evolution of live-attenuated YFV-17D virus was also monitored when the virus was launched as molecular clone from a plasmid (herein called PLLAV, plasmid-launched live-attenuated virus).

## Materials and methods

### Plasmids

Plasmid pShuttle/YFV-17D (WO2014174078A1) [29,30] is a derivative of pBeloBAC11 that carries a YFV-17D cDNA copy (17D-204 strain from ATCC, Genbank Genbank X03700.1) flanked at the 5’ by a SV40 promoter, and at the 3’ end by a hepatitis delta virus ribozyme. A second conditional origin allows for increased plasmid yields in *E. coli* EPI300-T (Epicentre) [31]. When used for immunization, pShuttle/YFV-17D is dubbed Plasmid-Launched Live-Attenuated Virus Vaccine (PLLAV). PLLAV was formulated for i.p. injection in 33% v/v 1,2-propanediol containing 17% w/v calcium carbonate microflowers as previously described [32].

### Virus stocks

YFV-17D was derived from the commercial YFV-17D vaccine (Stamaril®, Sanofi-Pasteur MSD, Brussels, lot H5105). For the generation of virus stocks, 10^6^ BHK-21J cells [33] were inoculated with 100 µL of Stamaril® diluted into 20 mL MEM/2% FBS, incubated for 1 h at room temperature, and incubated with 40 mL fresh medium at 37 °C, 5% CO_2_ for viral growth. After 7 days, virus-containing supernatant was harvested by centrifugation at 400xg for 10 min and stored at −80 °C for further use. The infectious content was determined by plaque assay (*see infra*) with one plaque-forming unit (PFU) equalling 10–100 median cell culture infectious doses (CCID_50_), The infectious content was determined by plaque assay (see infra) with one plaque-forming unit (PFU) equalling 10–100 median cell culture infectious doses (CCID50) [approximately 10 International Units (IU) [34] although virus stocks were not back-titrated against the WHO potency standard (NIBSC Code 99/616)].

### Mice and tissue samples

Brains sections of AG129 mice [35] that had been inoculated intraperitoneally with either 10^4^ PFU (Brains 2, 3, and 4), 10^−1^ PFU (Brains 9 and 10), 10^−2^ PFU (Brain 11) of YFV-17D derived from Stamaril®, or 20 µg of PLLAV-YFV-17D (Brains 5, 6, 8, 12, 13, 14, and 15) were stored frozen at −80 °C. Mice were euthanized as soon as overt signs of viral encephalitis (sudden weight loss, hindlimb paralysis, hunched posture) were observed. YFV-17D RNA was detected by quantitative RT–PCR using primers and probes (Table S3) as decribed [36].

### Genome amplification

Viral genomes extracted from cell culture supernatants (NucleoSpin RNA, Macherey-Nagel) and brain sections (RNeasy Mini, Qiagen) were amplified by RT–PCR (qScript One-Step RT–PCR, Quanta Bioscience) as overlapping 2 kb amplicons using primers (Table S1) as described [37]. RT–PCR conditions were as follows: RT at 48°C for 20, 3 min at 95°C, and 40 cycles of at 94°C for 30 s, at 60°C for 30 s, and at 72°C for 2 min, and final extension at 72°C for 10 min. Amplicons were gel purified and subjected to direct Sanger sequencing (BigDye v3.1).

### Sequence analysis

For de novo assembly of viral genomes, sequences were submitted to SeqScape v2.6, trimmed from both ends and aligned to the reference YFV-17D genome (Genbank X03700.1) (Supplementary Table S6), excluding reads with low quality and/or few nucleotides (less than 200 bp). Final alignments were corrected manually. Mega X [38] was used for phylogenetic analysis and to construct phylogenetic trees.

Full-length consensus genomes generated for (i) Stamaril® (lot H5105), (ii) for the vaccine passaged once and twice on BHK-21J cell (reflecting viral amplification and adaptation during plaque purification), and (iii) of representative clones of major *bona fide* variants present in Stamaril® were submitted to NCBI-Genbank with accession numbers MN708488 — MN708497 (Supplementary Table S7).

### Measurement of absolute diversity

Viral diversity was measured by (i) the mutation frequency (mutant clones divided by the total number of clones analyzed) (ii) the Shannon entropy [39–41], (iii) the Simpson index of diversity (1-D) [42] and (iv) the Hamming distances [43–45]. Shannon entropy of each brain was calculated using the following formula [40]:

$$s_{n}=-\frac{\Sigma_{i=1}^{n}f_{i}(\ln f_{i})}{N}$$

Where *n* is the number of different species identified, *fi* is the observed frequency of a particular variant in the quasispecies, and *N* is the total number of clones analyzed [40,41,46]. The Simpson index of diversity was calculated as:

$$D=\frac{\sum \;n(n-1)}{N(N-1)}$$

Where n is the total number of variants of a particular species, and N the total number of variants in the population.

### Plaque assay and plaque purification

Serial dilutions (1/50–1/31250) of brain homogenates (in MEM/ 2% FBS) were added to 10^6^ cells/well of BHK-21J cells grown in 6-well plates. Virus was removed after 1 h at 37°C, cells washed 3 times with PBS, and overlaid with 3 ml of 0.5% low melting agarose (Invitrogen) in MEM/2% FBS. After 7 days at 37°C for, cells were stained with 1% MTT (3-[4,5-Dimethylthiazol-2-yl]−2,5-Diphenyltetrazolium Bromide) at room temperature for up to 1 h. Agar from visible plaques were punched out randomly, disregarding any differences in plaque phenotype (size or colouring), dissolved in 1 mL of MEM/2% FBS by vortexing, followed by centrifugation at 2000xg for 5 min. For virus amplification, 500 µl of plaque supernatant was used to infect 10^6^ BHK-21J cells in 6-well plates. At day 4 post infection, virus supernatants were harvested and stored at −80°C for RNA isolation.

### Statistical analysis

All data were analyzed using GraphPadPrism v7 or R [45] and results expressed as mean values ± standard error of mean (SEM). Comparison between groups was performed using Mann–Whitney test with Bonferroni’s correction, and *p*-values <0.05 were considered statistically significant. *P = 0.05; **P < 0.01; ***P < 0.001; ****P < 0.0001, ns = not significant.

## Results

### Genetic stability of YFV-17D and plasmid-launched YFV-17D following inoculation of AG129 mice.

**Figure 1.**
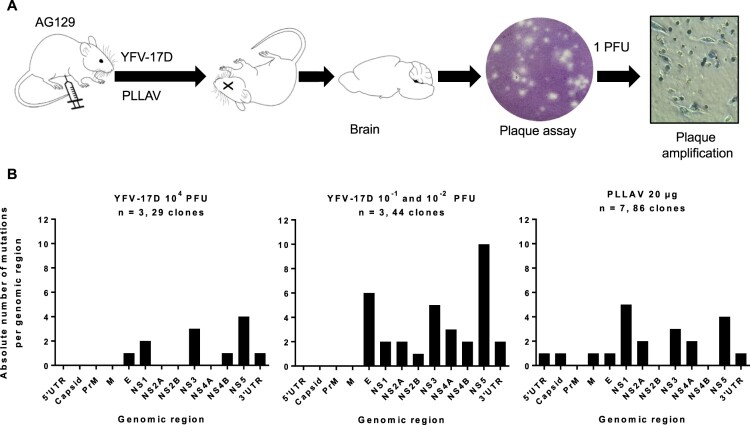
Mutation spectrum of YFV-17D and PLLAV following infection in AG129 mice. (A) Schematic presentation of study design. AG129 mice were inoculated i.p. with either a range of inocula of YFV-17D; 10^−2^–10^−1^ PFU, or 10^4^ PFU of YFV-17D (*n* = 3/group) or with 20 µg of PLLAV (*n* = 7). Mice brains were harvested and homogenized for plaque purification prior to sequencing. (B) Mutation frequencies in brain-derived YFV-17D and PLLAV following neurotropic infection in AG129 mice.

To investigate the evolution of YFV-17D and plasmid-launched YFV-17D in mice, six to eight weeks old AG129 mice were inoculated intraperitoneally with either 10^−2^ (corresponding to ∼1 CCID_50_), 10^−1^ or 10^4^ PFU (> 10^5^ CCID_50_) of YFV-17D, or with 20 µg of the PLLAV-YFV-17D plasmid (*n* = 2, 1, 3, and 7, respectively); infectious titres and viral RNA loads in mouse brains at the time point of euthanasia (when mice presented with signs of encephalitis, such as hunched posture, limping and paralysis) were determined by plaque assay and qRT-PCR, respectively (Supplementary Figure S1). One out of 2 mice inoculated with 10^−2^ (corresponding to ∼1 CCID_50_) did not develop any overt signs of disease, had no detectable viruses in the brain, nor seroconverted (data not shown), and was excluded from further analysis. For each brain at least *n* = 6 (6–15) individual plaques (Supplementary Table S1) were independently expanded and subjected to direct Sanger sequencing (Figure 1A). In total, 73 full virus genomes from the brains of six mice that had been inoculated with YFV-17D were analysed, each comprising in total 10862 nucleotides and collectively resulting in the identification of 46 distinct nucleotide changes as compared to the consensus sequence of Stamaril® that had been used as the virus inoculum (Supplementary Table S4). Generally, the nucleotide variants observed in the individual plaque-purified virus clones were scattered along the E, NS1, NS2A, NS3, NS4B, NS5 and 3’ UTR genomic regions (Figure 1B, Table 1). No mutations were observed in the 5’ UTR and capsid genes of one YFV-17D variants (Figure 1B, Table 1). This equal distribution suggests that there are no hotspots for mutations in the YFV-17D genome during replication and neuroinvasion in mice: the observed mutation rate per gene was comparable (between 10^−4^ and 10^−3^ substitutions per gene; Table 1) with no significant differences among all condition neither for Shannon nor Simpson entropies (*p*-values >0.05, Mann–Whitney), and hence no evidence for the selection of mutations that could be linked to neurotropism or neurovirulence [48]. The overall nucleotide substitution rate per viral genome was between 10^−4^ and 10^−5^, in support of the relatively low mutation rates reported for yellow fever vaccine virus [36,49] as compared to other RNA viruses [50,51], especially considering the prolonged time between inoculation and euthanasia (13–18 days, median = 16 days) available for expansion of the virus populations.

**Table 1.** Nucleotide substitutions per genomic region of YFV-17D versus PLLAV

Gene	Mutation rate		Mutation rate	
	# of Nucleotide changes*	YFV-17D	# of Nucleotide changes	PLLAV
5’ UTR	0	0	1	8.47E × 10^−3^
Capsid protein	0	0	1	2.75E × 10^−3^
prM/M	1	4.06 × 10^−3^	1	4.06E × 10^−3^
E	7	4.73 × 10^−3^	1	6.8E × 10^−3^
NS1	4	3.34 × 10^−3^	5	4.0E × 10^−3^
NS2A	3	6.0 × 10^−3^	2	4.0E × 10^−3^
NS2B	1	2.56 × 10^−3^	0	0
NS3	8	4.81 × 10^−3^	3	1.16 × 10^−3^
NS4A	3	3.48 × 10^−3^	2	2.32 × 10^−3^
NS4B	3	8.93 × 10^−3^	0	0
NS5	14	5.15 × 10^−3^	4	1.47 × 10^−3^
3’ UTR	3	5.89 × 10^−3^	1	1.96 × 10^−3^

*Sum total of all nucleotide changes observed in the virus population regardless of YFV-17D dose administered. Mutation rates were calculated by dividing the number of mutations observed in each genomic region by the number of nucleotides that constitute the genomic region. Pairwise comparison of the Shannon and Simpson entropy per genome region for each of the conditions (Stamaril® low and high, PLLAV) revealed no significant differences in entropy between regions (*p*-values >0.05, Mann-Whitney test).

**Figure 2.**
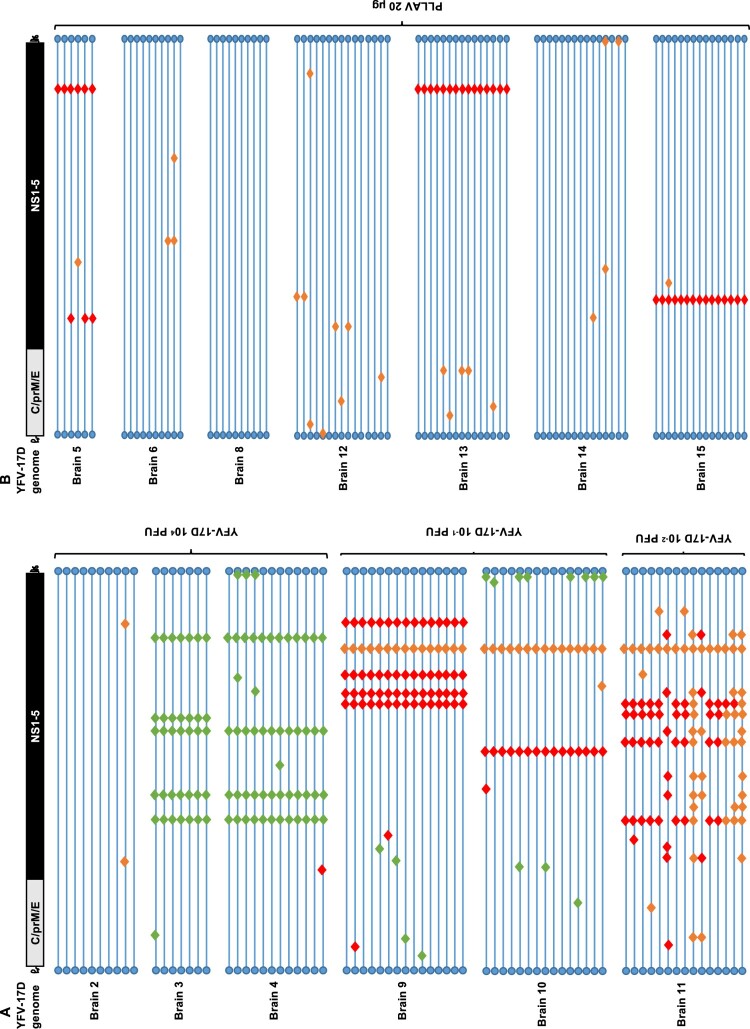
Mutation patterns observed in YFV-17D infected mice. (A) Graphical presentation of genomes of 73 brain-derived, plaque-purified virus clones of YFV-17D following i.p. inoculation and neuroinvasion in AG129 mice (*n* = 6). (B) Graphical presentation of 86 similar virus clones following i.p. injection of 20 µg of PLLAV-YFV17D and neuroinvasion in AG129 mice (*n* = 7). Horizontal blue lines represent individual YFV-17D genomes with blue circles as 5’ and 3’ UTRs, respectively. Red, green and orange diamonds represent missense, silent and mixed mutations, respectively (mixed = parental + mutant nucleotide). For a full list of mutations see Supplementary Tables S4 and S5.

**Figure 3.**
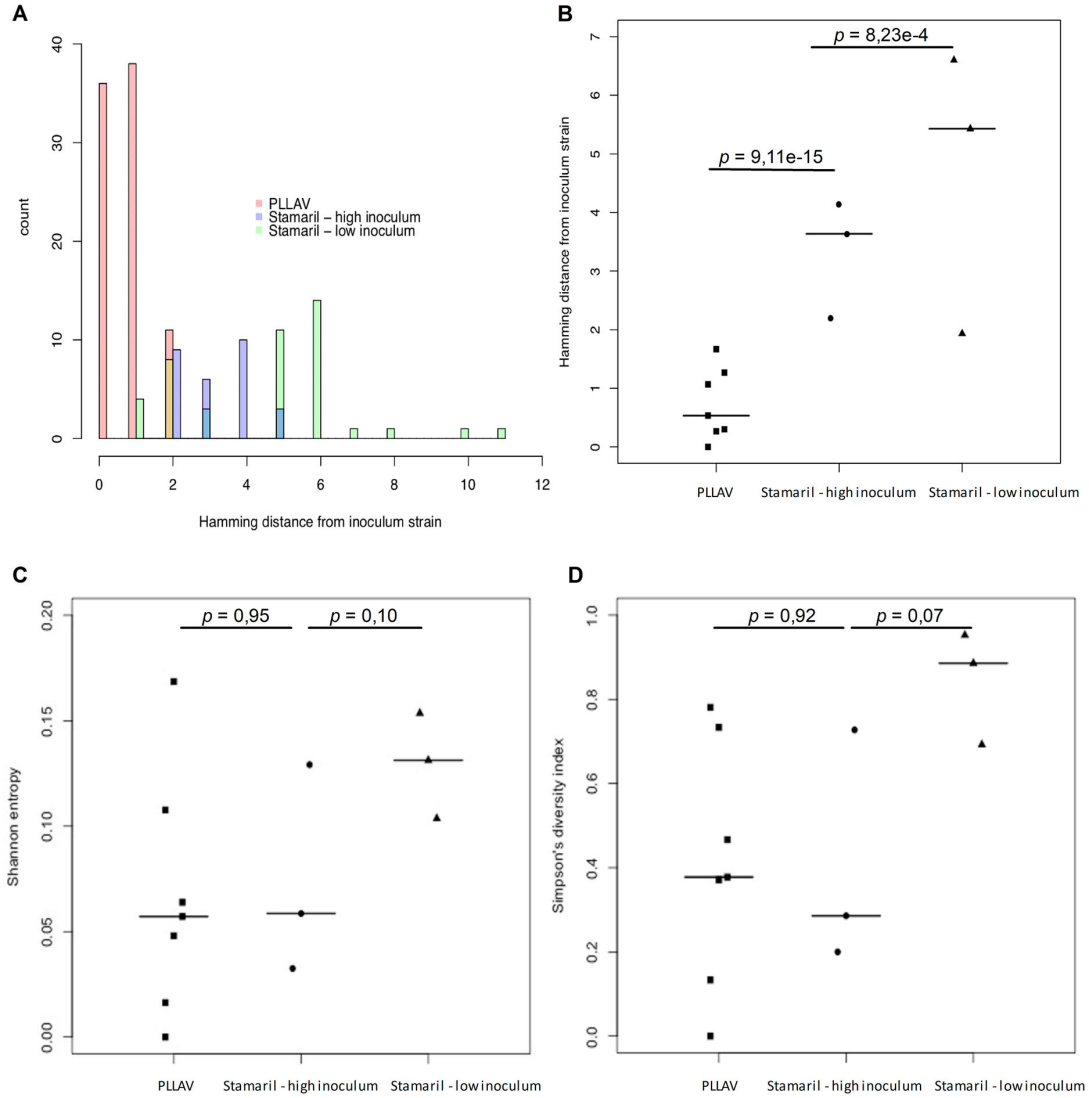
Diversity of virus variants in brains of mice inoculated with YFV-17D and PLLAV. (A) Hamming distances calculated for 10^4^ PFU [high inoculum] (*n* = 3, 29 clones), 10^−1^ PFU + 10^−2^ PFU [low inoculum] (*n* = 3, 44 clones) of YFV-17D, and PLLAV (*n* = 7, 86 clones). (B) Hamming distances computed for each virus inoculum. (C) The Shannon entropy and (D) Simpson’s index of diversity for each inoculum. Data for PLLAV are from 2 independent experiments (*n* = 3 each), bars presented median values. Student t-test for statistical comparison between groups.

No mutations were observed in 12% (9/73) of brain-derived YFV-17D clones, while the remaining 88% (64/73) carried at least one mutation (Figure 2). Viruses with no mutations in their genomes were exclusively isolated from a mouse that was injected with the high(est) inoculum, 10^4^ PFU (Figure 2A, Brain 2). In the brain of mice that had been injected with a low titre of the vaccine virus, increased mutation rates were noted (Figure 3A,B); the highest number of mutations were recorded in a mouse that had been inoculated with 10^−2^ PFU of Stamaril®-derived YFV-17D (Figure 2A, Brain 11).

As was the case for the YFV-17D infected mice, in mice that had been inoculated with PLLAV-YFV-17D, nucleotide changes observed in brain-derived viruses were scattered throughout the E, NS1, NS2A, NS3, NS4A and NS5 regions **(** Figure 1B, Table 1**)**. Forty-two percent (42%) (36/86) of virus clones that were plaque-purified from the brain of mice that had been inoculated with PLLAV-YFV-17D had no mutations (Figure 2B) (in contrast to 12% observed in YFV-17D inoculated mice, Figure 2A) whereas the remaining 58% (50/86) had either only 1 or 2 mutations (median = 1) per genome. Hence the incidence of nucleotide variants in the PLLAV-YFV-17D derived viruses was markedly lower compared to the frequency of variant clones that were obtained from mice that had been injected with YFV-17D (Stamaril®); where 0–11 mutations per genome (median = 5) were observed (Figure 2B).

### Impact of the YFV-17D inoculum size on evolution of the virus in AG129 mice

It was next investigated whether the YFV-17D population diversity may be a function of inoculum size and differed in mice that were infected with a (very) low inoculum of the vaccine virus instead of a high viral dose. Importantly, the pathogenesis induced by YFV-17D was highly similar and independent of the inoculum size, as confirmed by similar viral brain RNA loads at euthanasia (Supplementary Figure S1) and no obvious changes in plaque phenotype in viruses isolated from respective mouse brains (not shown). Nevertheless, neuroinvasion and progression to neurotropic disease took longer in mice in the low inoculum group; surviving on average longer (16–18 days to euthanasia) compared to the high inoculum group (13–16 days to euthanasia; no significant difference). As a very low inoculum may allow the maximal expansion of the populations and thus a maximal likelihood to accumulate mutations, data from mice injected with either a high (10^4^ PFU) or low (10^−1^ or 10^−2^ PFU) inoculum of YFV-17D were directly compared. Data from mice inoculated with 20 µg of PLLAV-YFV-17D and using a molecular clone of YFV-17D were included as benchmark. Diversity was measured by Hamming distance, Shannon entropy and Simpson diversity index (Figure 3A–D).

The Hamming distance [43–45] **(** Figure 3A,B**)** measures the number of nucleotide substitutions in viral genomes and group variants based on the number of substitutions when compared with the reference sequence (GenBank X03700). Clones with the same number of mutations are grouped together. The type of mutation or its position in the genome is not taken into consideration in this analysis. A significant difference in Hamming distances between the high and low titre YFV-17D inocula were noted (*p* < 0.001). The highest individual diversity was observed in one particular mouse injected with as little as 10^−2^ PFU (∼1 CCID_50_). For further analysis, the clones obtained from the brain of this mouse (*n* = 15) were grouped with those retrieved from two mice that had been inoculated with 10^−1^ PFU of the vaccine virus (together collectively named the “low inoculum”). Clones from the low inoculum group had between 1 and 11 nucleotide substitutions per genome (Figure 3A). Supportive, though not significantly different, there was a consistent trend for a greater diversity in the low inoculum group also regarding Shannon entropy (Figure 3C) and Simpson diversity index **(** Figure 3D**)**. A higher mutation rate in the low inoculum group could most likely be attributed to the increased number of replication cycles of the inoculated virus. Regardless of the titre of the inoculum, the mutation rates of the virus clones did not exceed 10^−4^ substitutions per genome, underscoring the stability of the YFV-17D genome.

### YFV-17D passes through a bottleneck during neuroinvasion in AG129 mice

**Figure 4.**
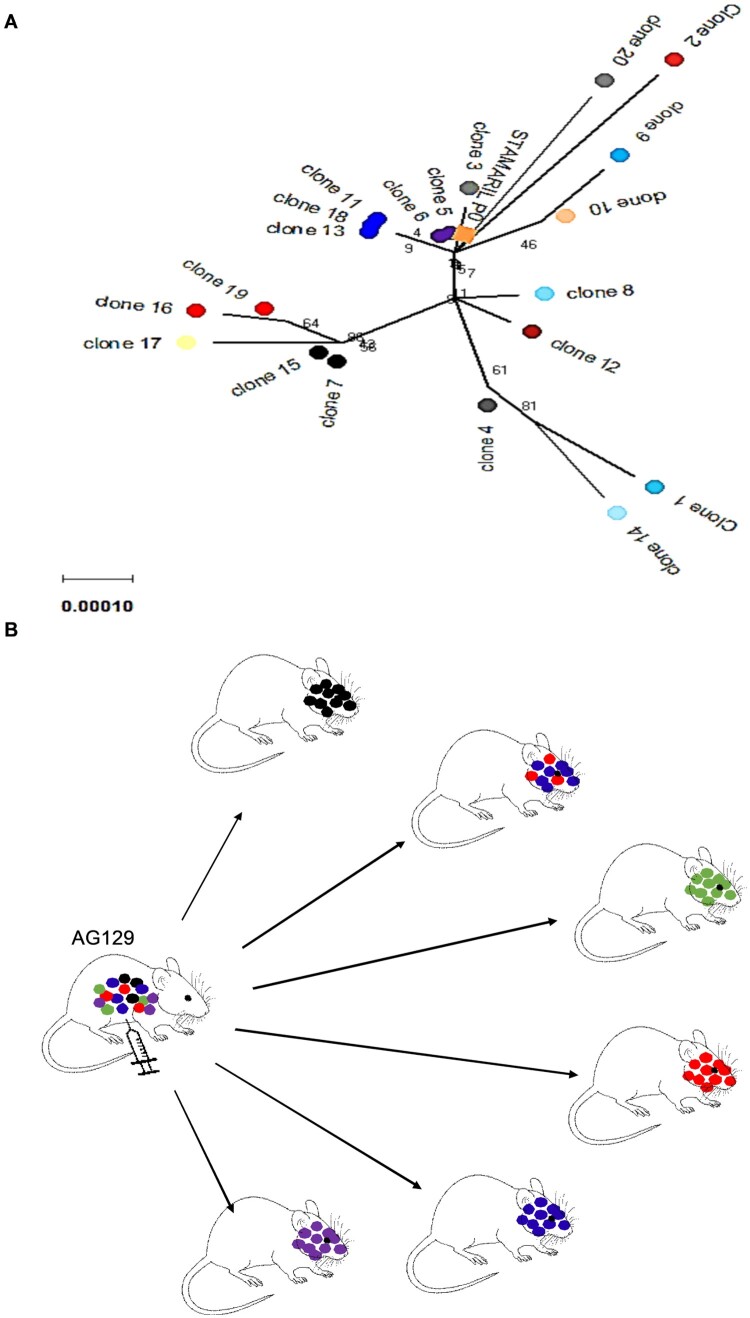
Evolutionary analysis and diversity of YFV-17D before and after inoculation in AG129 mice. (A) Diversity in plaque-purified virus clones (*n* = 20) of Stamaril® (lot H5105) prior to injection in mice. A consensus sequence (Stamaril® P0) was generated from 20 plaque-purified virus clones. Evolutionary analysis in MEGA X [36] using Maximum Likelihood method and Kimura 2-parameter model [51]. Tree by Neighbor-Join and BioNJ algorithms to a matrix of pairwise distances (Maximum Composite Likelihood estimates), selecting the topology with superior log likelihood value (−15353.23). Branch lengths drawn to scale (scale bar: 0.0020 substitutions per site). The analysis involved *n* = 21 nucleotide sequences, including GenBank X03700 as reference genome, with a total of 10862 positions in the final dataset. (B) Graphical visualization of genetic diversity and segregation observed in YFV-17D populations following neurotropic infection in AG129 mice.

**Figure 5.**
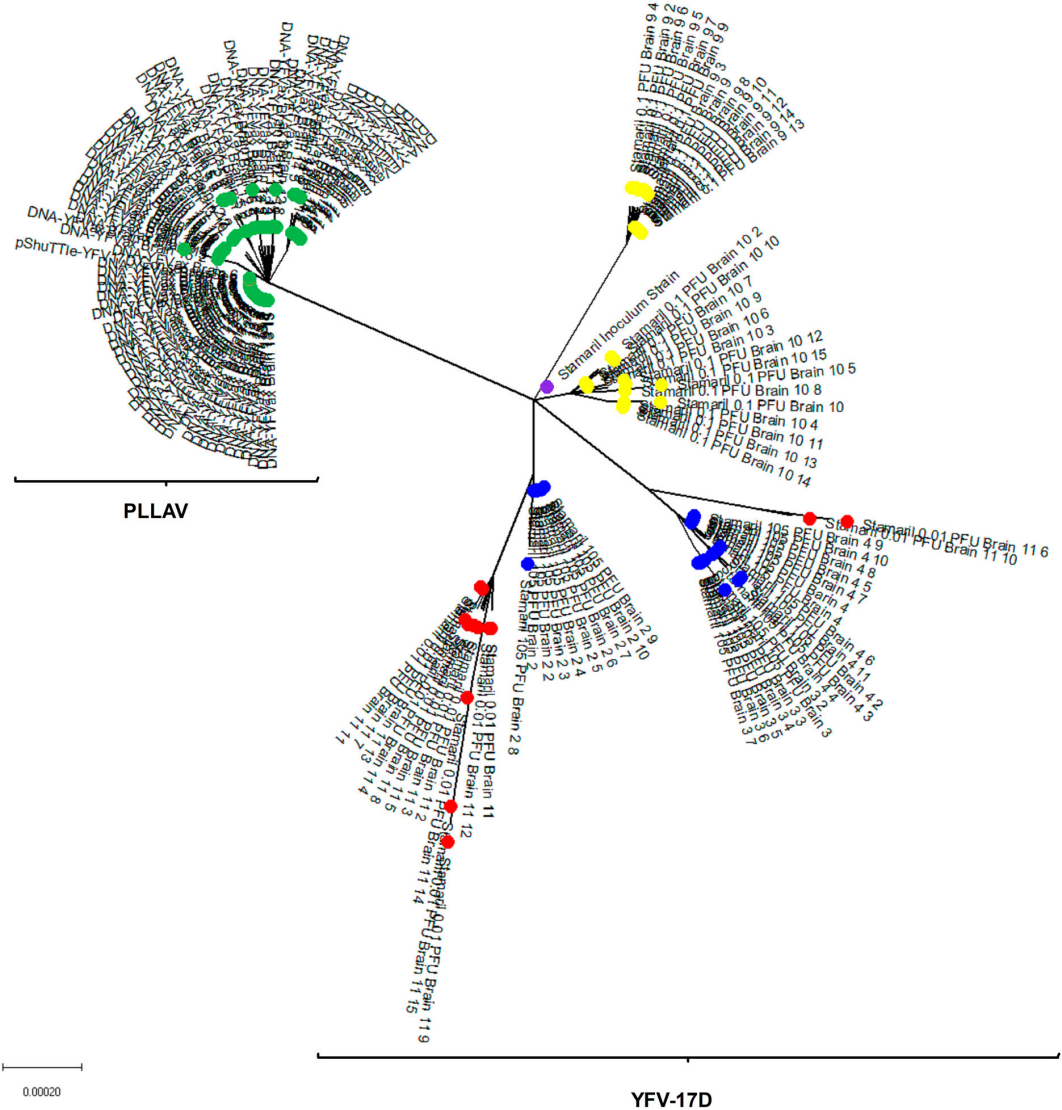
Phylogenetic tree of Stamaril® and PLLAV-derived YFV-17D clones from AG129 mice. Coloured dots represent the different inocula. Green: PLLAV 20 µg; blue dots: 10^4^ PFU Stamaril; yellow: 10^−1^ PFU Stamaril; red: 10^−2^ PFU Stamaril: purple: consensus sequence (P0) of Stamaril®. Evolutionary history inferred using the Neighbor-Joining method [52]. The optimal tree (sum of branch length = 0.00785397) drawn to scale. Evolutionary distances (in number of base substitutions/site) computed using the Kimura 2-parameter method [17], involving *n* = 162 nucleotide sequences (*n* = 10862 positions in the final dataset after pairwise deletion of ambiguous positions).

For the poliovirus, it has been demonstrated that bottlenecks restrict the spread of viral variants from the injection site to the brain [18, 19]. We set out to explore whether a similar bottleneck exists for YFV-17D. To that end, the infection model in AG129 mice is ideally suited because it allows for sufficient replication and trafficking of the virus from the injection site (i.p.) to (also) the brain. Prior to inoculation in AG129 mice, the initial YFV-17D (Stamaril®) virus stock was characterized by Sanger sequencing (Supplementary Figure S2). In addition, virus clones (*n* = 20) were plaque-purified from BHK-21J cells that had been infected with Stamaril® and were amplified once on BHK-21J cells prior to whole viral genome sequencing (Figure 4A). Both plaque-purified Stamaril® clones (Supplementary Table S4) and plaque-purified YFV-17D clones from mouse brains (Figure 5A, B, Supplementary Figure S3) appear to be highly heterogeneous (different variants) (Supplementary Table S5). By comparing the sequences of 20 different randomly picked YFV-17D clones plaque-purified from Stamaril® directly *in vitro* (Figure 4A), we hypothesize that Stamaril® comprises at least 5 (major) virus variants, and the dominant virus variants isolated subsequently from infected mouse brains seem to an large extent to pre-exist in the heterogeneous Stamaril® population (Figure 4B, Supplementary Tables S7 and 8). The clonal diversity in the particular Stamaril® lot analysed in this study translated into a Simpson *1-D* index of about 0.042, in line with an obvious diversity previously observed by others when either comparing molecularly cloned cDNA fragments of Stamaril® [52], or deep sequencing of another 17D-204 vaccine [20], that yet escapes detection in consensus sequences [28]. In each of the brains, one species appeared to dominate. Only in the brain of one mouse (that had been inoculated with 10^−2^ PFU) (Figure 2, Brain 11) 2 major variants were detectable (Figure 4B and Supplementary Figure S3). By contrast, in mice that had been injected with 20 µg of plasmid encoding a molecular clone of YFV-17D, the virus replicates to low diversity and clustered as one homogenous virus species, with no separate clonal linages of YFV-17D arising when comparing individual brains (Figure 5, Supplementary Figure S4). Importantly, no obvious selection for more aggressively growing virus variants could be observed neither *in vivo*, nor regarding plaque phenotype *in vitro* of the individual virus variants isolated from mouse brains after neurotropic replication (Supplementary Figure S5). In conclusion, in mice that had been inoculated with YFV-17D, the diversity observed in brain-derived YFV-17D clones (Figure 3A-D and Figure 5) is hence likely the consequence of pre-existing heterogeneity in the YFV-17D inoculum (i.e. Stamaril®) prior to injection (Figure 4A, Supplementary Figure S3). Diversity stays very low if the inoculum consists of a molecular clone of YFV-17D.

## Discussion

The primary goal of the study was to assess the genetic stability and diversity (mutation rates and patterns) arising during YFV-17D replication in a vertebrate host. We chose to analyse populations by sampling and direct sequencing of representative numbers of plaque-purified full virus genomes. This allowed to assess and compare clonal sequence variability arising during error-prone viral replication *in vivo* of the licensed YFV-17D vaccine.

The two main substrains of YFV-17D currently commercialized as vaccines are YF17D-204, and YF17DD [17]. The evolution of YFV-17D live-attenuated virus was monitored when it was either injected as a viral inoculum directly derived from Stamaril® or when the viral genome was launched as a clonal virus from a plasmid (PLLAV-YFV-17D, 17D-204 ATCC strain). As the vertebrate host, we used IFN α/β and γ-receptor deficient AG129 mice; these animals are hypersusceptible to infection with either YFV or YFV-17D [55–57]. Inoculation of these mice with YFV-17D (equivalent to a fractional dose of Stamaril®) results, on average after 12 days, in a lethal disseminated and neuroinvasive infection. When very low inocula are being used disease onset and mortality is delayed, the mean day to euthanasia is then on average 16 days [57]. This model thus allows lengthy replication and expansion of the initial (minimal) inoculum and is thus ideally suited to study evolution of the viral population. Hamming distances, Shannon entropy values and Simpson’s indices of diversity reveal a relatively low viral diversity in mice inoculated with a rather high inoculum (10^4^) PFU of YFV-17D (Figure 3). The diversity increases with decreasing titres of the inoculum. We observed the highest diversity in a mouse injected with a very low inoculum (10^−2^ PFU, corresponding to ∼1 CCID_50_) of YFV-17D. Virus isolates from the brain of this mouse comprised a mutant spectrum of at least eight different virus variants (out of 15 brain-derived isolates) that arose from the parental Stamaril®. Virus isolates from the low titre inoculum had increased Hamming distances significantly different (*p *< 0.0001) compared with isolates from the higher titre inoculum. In line with this finding, the Shannon entropy and the Simpson’s index of diversity were also higher for the low titre compared to the high titre inoculum. This is unexpected considering that YFV is thought to reach the brain through the blood brain barrier [58] whereby an increase in the titre of the inoculum may possibly increase the number of virus variants that would eventually reach the brain. A possible explanation may thus be that the viruses present in the lowest titre inoculum (< 1 PFU) may have replicated and evolved to generate highly replication competent viruses; thereby possibly selecting for variants with increased neuroinvasive and neurovirulent properties. Though no direct experimental evidence for this hypothesis could be provided in this study, at least an increased infectious virus yield (yet not viral RNA load) could be isolated from the brains of the low versus the high titre infected groups (Supplementary Figure S1B). The mice that had received the low inoculum lived on average a couple of days longer than those that had been injected with a high titre inoculum [57]. A longer lifespan may also allow more replication cycles/expansion of the population, thereby increasing the likelihood of the acquisition of novel mutations. It is worth noting that variants that were originally present as minorities in the heterogeneous Stamaril® population emerged to be dominant variants orchestrating infection and viral pathogenesis in some mice. This observation is further suggestive for a viral bottleneck-mediated quasispecies restriction [19] and subsequent evolution of this variants. It has been reported that YFV does not experience bottlenecks during its replication in interferon type I (IFN α/β) deficient mice [59]. However, as reported here (in an infection model wherein each variant in the population has the potential of infecting and causing disease), the population bottleneck may be a plausible phenomenon to explain the neuroinvasion of the mouse brain by a single or at most a few variants, in particular variants that existed already in the inoculum as minorities. These findings may have implications for WHÓs recent endorsement of using fractional dosing to cope with vaccine shortage during recent large emergency of YF epidemics (see *infra*) [60].

To study the evolution of an expanding YFV-17D population, yet starting from a homogenous inoculum, we launched the virus as a clonal genome from a plasmid using our PLLAV technology [29]. From the low diversity observed in these mice **(** Figure 2B**)**, there is no assumption to believe that launching of YFV-17D replication from PLLAV-YFV-17D (driven by the cellular RNA polymerase II) would add extra variability to the circulating vaccine virus population [61,62]. In our mouse model, PLLAV-launched YFV-17D had the lowest diversity indices **(** Figure 3A,B**)**; in fact, 42% of the clones derived from the brain had no mutations in their genomes whereas the remaining 58% carried only 1–2 (mostly silent) mutations. This is in stark contrast to the situation where mice had been injected with the vaccine virus (quasispecies); in that case, YFV-17D clones carried up to 11 nucleotide variants per genome **(** Figure 2A). Clones obtained from one of the PLLAV-YFV-17D inoculated mice had a Shannon entropy and Simpson index of diversity of zero, indicating that YFV-17D can replicate without errors during broad expansion in the vertebrate host. This finding is contrary to the popular paradigm that virus pathogenesis increases with increasing viral diversity [21,62]. We here show that diversity is not strictly necessary for YFV-17D induced pathogenesis (the mouse without virus diversity in the brain had equally overt signs of infection/disease as mice carrying multiple variants). In addition, clones isolated from another mouse that had been injected with a low inoculum carried exclusively silent mutations that likely do not change the phenotype of the viruses (Supplementary Figure S5). Furthermore, only one dominant virus variant (could be isolated from each mouse brain (Supplementary Figure S3; exception Brain 11) despite the high diversity that pre-existed in the original YFV-17D (Stamaril®) inoculum, further corroborating that diversity does not directly correlate with viral pathogenesis.

Our findings may have important public health implications. The YF outbreaks in recent years (Angola, DRC, Nigeria, and Brazil) repeatedly depleted the YFV-17D vaccine stocks [60]. Consequently, WHO recommended fractional dosing (1/5) to meet the huge demands. Studies demonstrate that reducing the dose (of a sufficiently high tittered) YFV-17D does not affect immunogenicity and safety, however, the studies were restricted to adults [63–67]. Because of limited data in children, WHO expressed potential safety concerns and recommended full doses for children. WHO highlighted the urgent need for more studies on fractional YF doses in young children. Though our laboratory findings suggesting that YFV-17D accumulates only very few mutations, even after maximal expansion (following inoculation of an approximately < 1/10^5^ human dose) in highly permissive mice cannot directly be translated to the human situation in the field, our results suggest that the limited (one fifth) fractional dosing of YFV-17D may also in humans (most likely) not result in an increased risk of YEL-AND, at least not (to the experimental evidence provided by us and others studying the genetic stability during YFV-17D replication [20,49,68,69]) due to any apparent tendency of the vaccine virus to revert to or select for pathogenic variants. These results are also reassuring for the use of fractional-dose YF vaccination in children. A randomized non-inferiority trial was recently launched comparing seroconversion after fractional-dose in children (ClinicalTrials.gov number, NCT02991495), to provide more data on immunogenicity and safety of low inoculum dose vaccination in this vulnerable age group.

In conclusion, we confirm that the mutation rate of YFV-17D is very low, comprising between 10^−4^ and 10^−5^ nucleotide substitutions per genome following long term maximal *in vivo* replication in the vertebrate host. We provide evidence that pathogenesis of YFV-17D (and possibly other flaviviruses), does not necessarily correlate with virus diversity. The mutation frequencies, Shannon entropies, Simpson indices of diversity and the Hamming distances reveal that the plasmid-launched YFV-17D is at least as stable (but apparently obviously more stable), in immunodeficient mice, as the highly heterogeneous Stamaril® derived virus. We also report that fractional dosing in AG129 mice does not affect the stability and evolution of the YFV-17D vaccine. Overall, the high genetic stability of YFV-17D, even following maximal expansion in the vertebrate host (here immunodeficient mice) may largely lower the risks of antigenic drift or evolution of revertant virus vaccines.

## Supplementary Material

Supplemental Material
