# Supplementary material for: Limited evolution of the yellow fever virus 17d in a mouse infection model
Source: Emerg Microbes Infect. 2019 Dec 4;8(1):1734–46. doi: 10.1080/22221751.2019.1694394 (PMC6896426; doi:10.1080/22221751.2019.1694394)
Supplement: Supplemental Material [file TEMI_A_1694394_SM1471.docx]

RNA isolation

qRT-PCR

Plaque assay


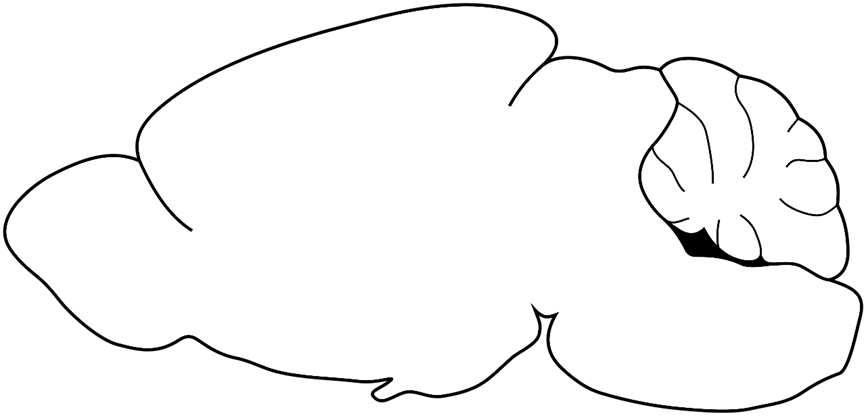


Mouse brain

YFV-17D


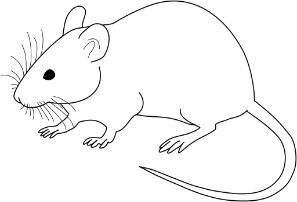

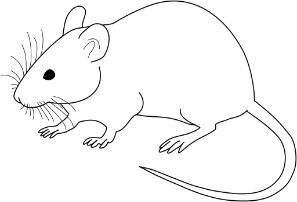

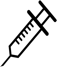


PLLAV

AG129

**A**

**B**

FIG. S1. Plaque assay and qRT-PCR on mice brains. (**A**) AG129 mice were inoculated with different doses of YFV-17D, namely; 10^-2^ - 10^-1^ PFU (low inoculum, n = 3), 10^4^ PFU (high inoculum, n = 3) of YFV-17D or PLLAV (n = 7). At euthanasia, mice brains were harvested, homogenized and the homogenate was used for plaque assay and qRT-PCR. **(B)** Plaque assay and qRT-PCR on brain homogenates following lethal infection. Data are presented as mean values with error bars indicating standard error of mean (SEM) (n = 3 animals analysed each per condition). Mann-Whitney two-tailed test with Bonferroni’s multiple corrections was used for statistical calculations. *P*-values < 0.05 were considered statistically significant. Horizontal dotted lines denote the limit of detection (L.O.D.) of the assay. *****P* < 0.00001, ns = no significant.


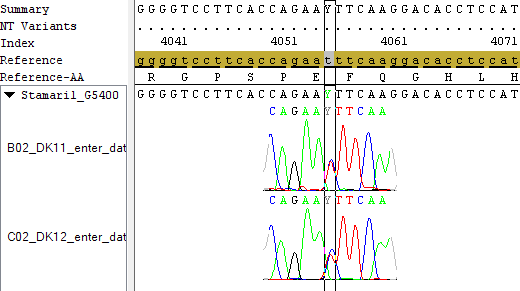

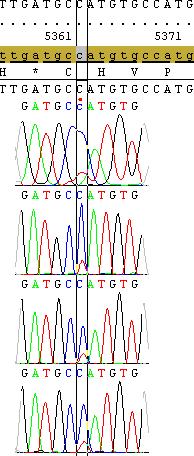

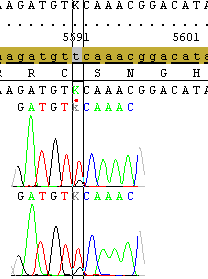

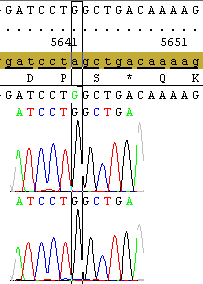

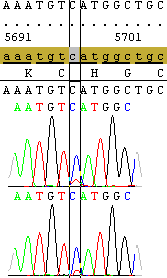

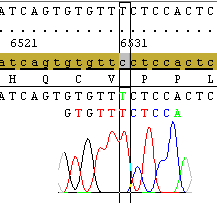

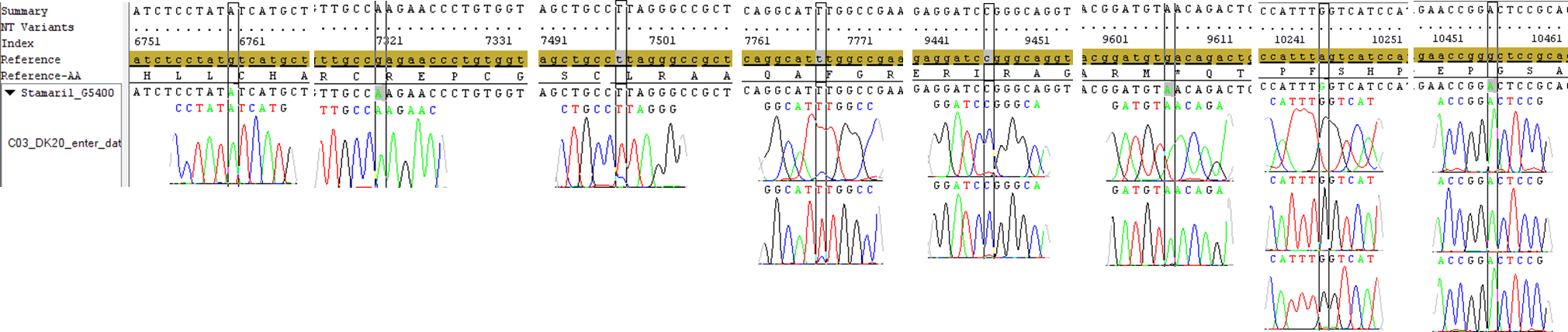


Fig. S2. Nucleotide differences and heterogeneities observed in Stamaril^®^ prior to inoculation in mice. The original Stamaril^®^ virus vaccine (lot H5105) was sequenced (direct Sanger sequencing of RT-PCR amplicons) prior to cell culture-amplification and inoculation in mice. A consensus sequence was generated from 20 plaque-purified virus clones (Fig. 4A and Fig. S3). Red arrow point to areas in the genome with heterogeneous (ambiguous) nucleotides herein referred to as mixed bases.


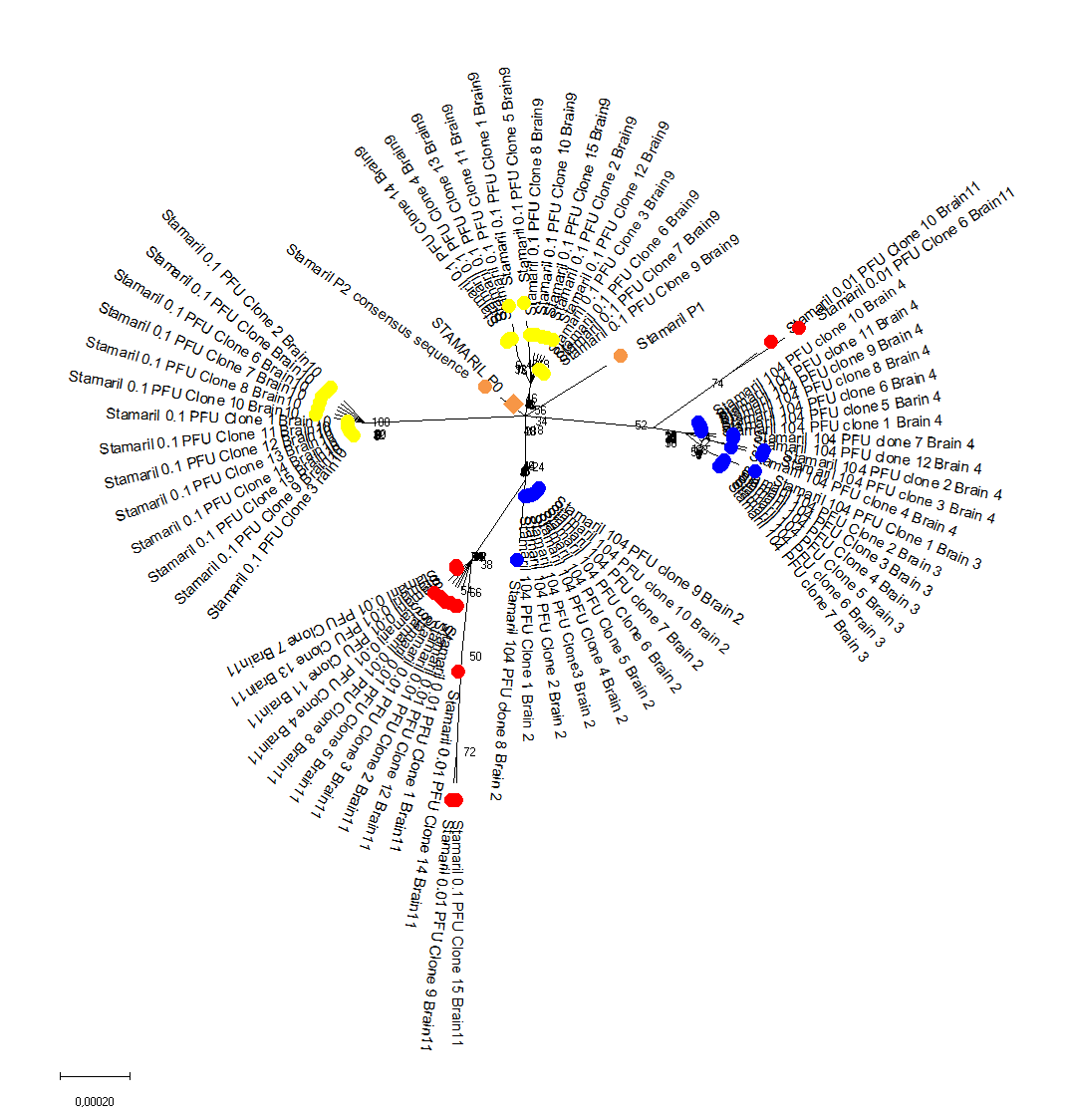


Fig. S3. Evolutionary analysis of YFV-17D clones from mice brains inoculated with vaccine virus derived from Stamaril^®^. The evolutionary history was inferred by using the Maximum Likelihood method and Kimura 2-parameter model (62). The tree with the highest log likelihood is shown. Initial tree(s) for the heuristic search were obtained automatically by applying Neighbor-Join and BioNJ algorithms to a matrix of pairwise distances estimated using the Maximum Composite Likelihood (MCL) approach, and then selecting the topology with superior log likelihood value. The tree is drawn to scale, with branch lengths measured in the number of substitutions per site. This analysis involved 76 nucleotide sequences. Codon positions included were 1st+2nd+3rd+Noncoding. There were a total of 10862 positions in the final dataset. Evolutionary analyses were conducted in MEGA X (55). Coloured dots represent the type/titers of inoculum injected in mice: Blue dots; 10^4^ PFU Stamaril, Yellow dots; 10^-1^ PFU Stamaril, Red dots; 10^-2^ PFU Stamaril, Orange; Consensus sequences of the original Stamaril^®^ inoculum passages 0, 1 and 2.


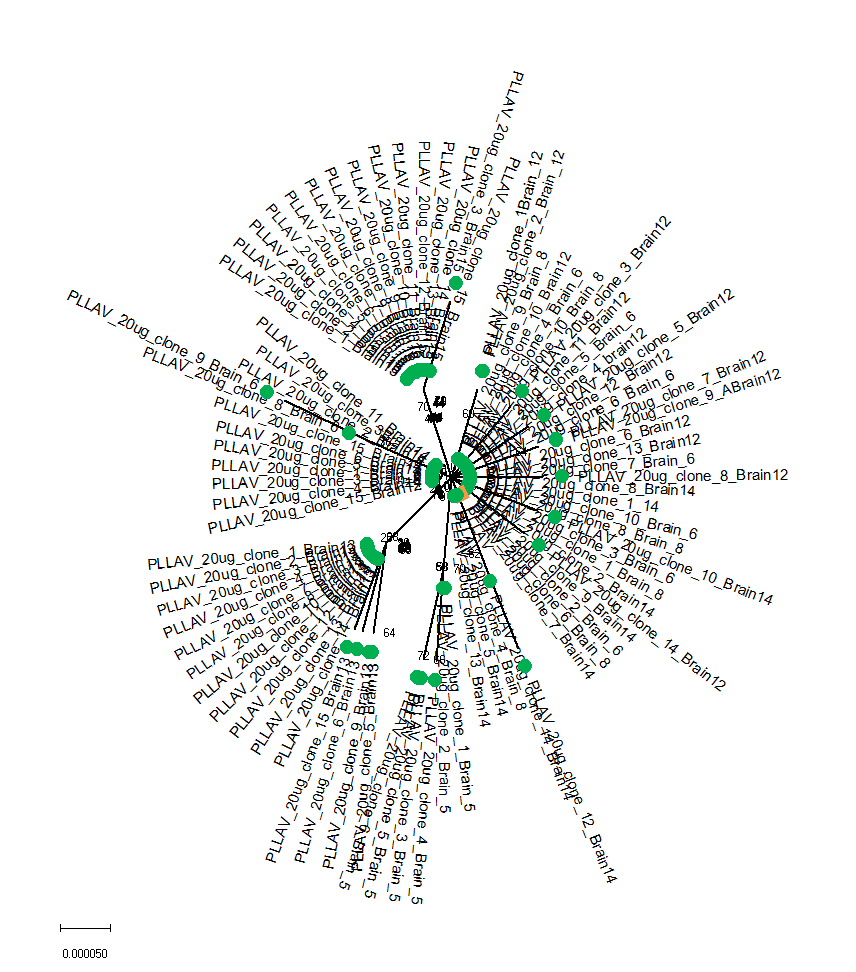


FIG. S4. Evolutionary analysis of YFV-17D clones from mice brains inoculated with PLLAV-YF17D. The evolutionary history was inferred by using the Maximum Likelihood method and Kimura 2-parameter model (62). The tree with the highest log likelihood is shown. Initial tree(s) for the heuristic search were obtained automatically by applying Neighbor-Join and BioNJ algorithms to a matrix of pairwise distances estimated using the Maximum Composite Likelihood (MCL) approach, and then selecting the topology with superior log likelihood value. The tree is drawn to scale, with branch lengths measured in the number of substitutions per site. This analysis involved 86 nucleotide sequences. Codon positions included were 1st+2nd+3rd+Noncoding. There were a total of 10862 positions in the final dataset. Evolutionary analyses were conducted in MEGA X (55). Coloured dots represent the dose of inoculum injected in mice: Green dots; PLLAV 20 µg, orange dot; Consensus sequence of the original PLLAV prior to inoculation.


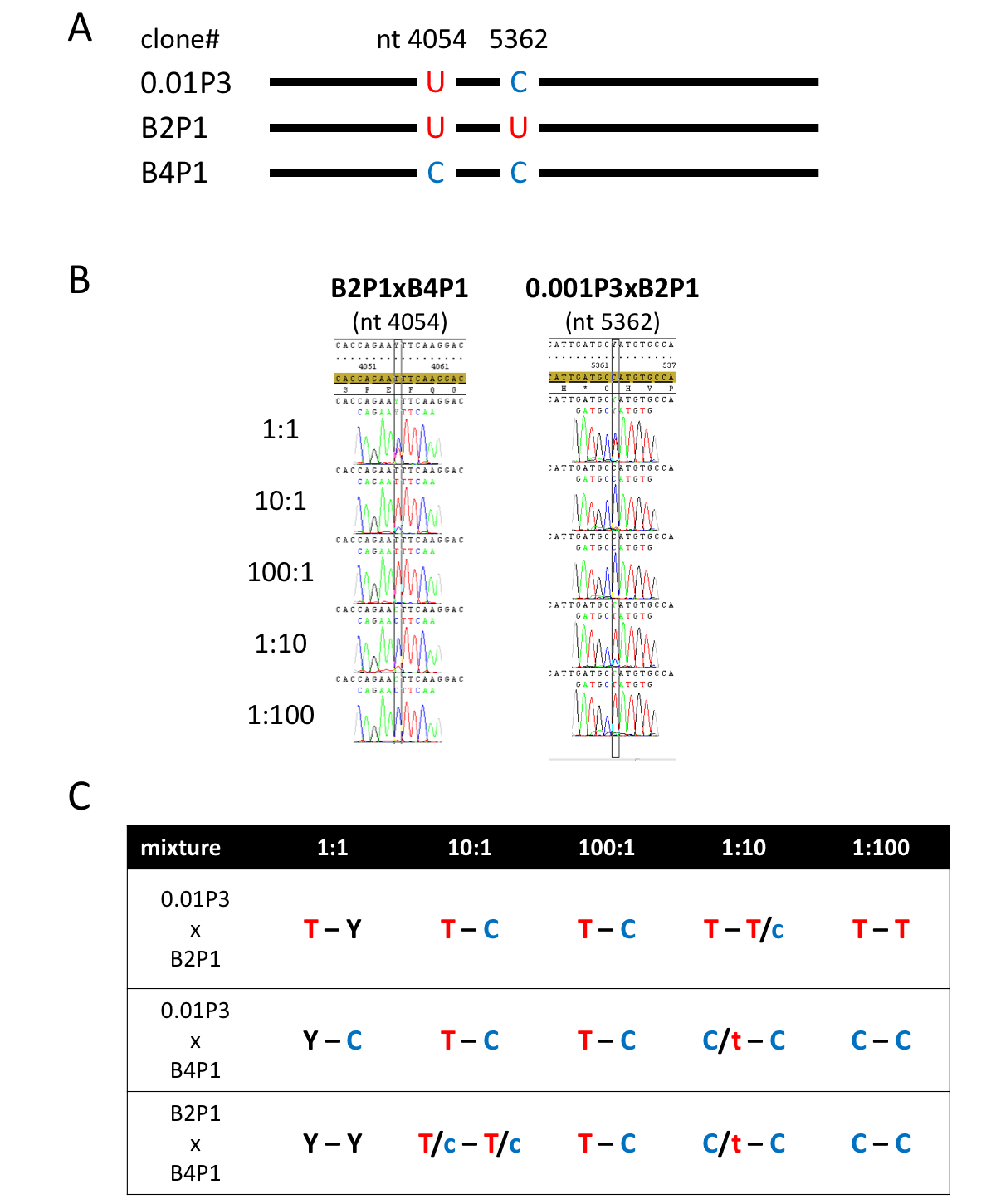


FIG. S5. Relative fitness of brain-derived YFV-17D clones. Assessment of competition between genetically distinct YFV-17D clones by direct sequencing of mixed virus populations. (**A**) Schematic representation of nucleotide variants (Supplementary Fig. S2) discriminating clones 0.01P1 (Stamaril 0.01PFU, plaque clone 1), B2P1 (10^4^ PFU Brain 2, plaque clone 1) and B4P1 (10^4^ PFU Brain 4, plaque clone 1) isolated from mouse brains after inoculation with YFV-17D (Stamaril^®^ lot H5150 derived virus). Each clone contained unique additional mutations (Fig. S3). (**B**) Plaque purified viruses were mixed in varying ratios and passaged twice on BHK-21J cells, followed by direct Sanger sequencing. Representative sequencing chromatograms are shown. (**C**) Relative peak height in sequencing tracks at nucleotide (nt) positions 4054 and 5362 observed at indicated ratios. Capitals indicating dominant and small letters minority nt species, respectively. At similar multiplicities of infection both virus variants co-exist for at least two passages, indicating an equal replication fitness. At higher mixing ratios, the respective minority variant is consistently lost from detection. C – cytidine; T – thymidine; U – uracil; Y – both C and T.

**Table S1 Samples analyzed for mutation complexity**

| **Sample** | **Passage history** | **Plaque purification** | **Analyzed in this study** |
| --- | --- | --- | --- |
| Stamaril^®^* | None | No purification | Full genomes |
| Stamaril^®^ | 1x on BHK-21J | 20 plaques | Full genomes |
| PLLAV | None | None  (direct sequencing of plasmid DNA) | Full genomes |
| YFV-17D | Brain 2 | 10 plaques | Full genomes |
|  | Brain 3 | 7 plaques | Full genomes |
|  | Brain 4 | 12 plaques | Full genomes |
|  | Brain 9 | 14 plaques | Full genomes |
|  | Brain 10 | 15 plaques | Full genomes |
|  | Brain 11 | 15 plaques | Full genomes |
| PLLAV | Brain 5 | 6 plaques | Full genomes |
|  | Brain 6 | 10 plaques | Full genomes |
|  | Brain 8 | 10 plaques | Full genomes |
|  | Brain 12 | 15 plaques | Full genomes |
|  | Brain 13 | 15 plaques | Full genomes |
|  | Brain 14 | 15 plaques | Full genomes |
|  | Brain 15 | 15 plaques | Full genomes |

* The original Stamaril^®^ (lot H5105) analyzed to generate the respective consensus sequences used in this study.

**Table S2 List of all primers used for sequencing in this study**

| Primer number | Primer Name/Orientation | Sequence 5’ – 3’ | Amplicon |
| --- | --- | --- | --- |
| **1036** | YFV-17D(+)1 Forward | AGTAAATCCTGTGTGCTAATTGAGGTG | A |
| 886 | YFV-17D(+)120 Forward | TGTCTGGTCGTAAAGCTCA |  |
| 1119 | YFV-17D(-)500 Reverse | TTTTCCGCACCAAGGTCACTCCA |  |
| 1120 | YFV-17D(+)700 Forward | CGCATATGGTAAGTGTGACTCA |  |
| 94 | YFV-17D(-)940 Reverse | GGCAATCACGACTCGTTGCG |  |
| 1097 | YFV-17D(+)1400 Forward | TTGCATGTAGGGGCCAAGCA |  |
| **1037** | YFV-17D(-)2005 Reverse | GATTGCCGCTGTAAGATCATCAG |  |
| **1038** | YFV-17D(+)1749 Forward | CTGGCGCAATGAGGGTTACAAA | B |
| 1098 | YFV-17D(-)2400 Reverse | CTCATGGACATTGTCATGTTTCT |  |
| 953 | YFV-17D(+)2500 Forward | AGATGGTATCTTCATATTTAGAG |  |
| 1100 | YFV-17D(+)3400 Forward | CACAATGCCGCCTGTGA |  |
| 1121 | YFV-17D(-)3100 Reverse | CAAGGTGTGGATCATCCATGT |  |
| 1102 | YFV-17D(+)3600 Forward | TGGTTGGAGGAGTAGTGCT |  |
| **1039** | YFV-17D(-)3725 Reverse | TTGAAAAGGCAGCAATCAACGC |  |
| 1040 | YFV-17D(+)3498 Forward | GGGTTACAGCTGGAGAAATACATGC | C |
| 954 | YFV-17D(-)3600 Reverse | ACATTTGCTTTGGTCCCTGTCT |  |
| 1122 | YFV-17D(+)3980 Forward | GTCACTATGGCTGAGGTGAGA |  |
| 345 | YFV-17D(+)5500 Forward | GGCAAATGAAAGTGCAAC |  |
| 342 | YFV-17D(+)4500 Forward | CTGCCCTCCATCCATTTGC |  |
| 344 | YFV-17D(+)5000 Forward | AACAGGAACGGAGAGGTG |  |
| **1041** | YFV-17D(-)5506 Reverse | ATTTGCCCTAGCTCTGTGCGCT |  |
| **1042** | YFV-17D(+)7278 Forward | GCCCCCACCAGGGTTGTTCTTTCT | D |
| 345 | YFV-17D(+)5500 Forward | GGCAAATGAAAGTGCAAC |  |
| 346 | YFV-17D(+)6000 Forward | ACTATTCTGAGCCTACAAG |  |
| 1103 | YFV-17D(+)6700 Forward | GGCCGGCTGTGGATATCT |  |
| 153 | YFV-17D(+)7001 Forward | GCCTGGACAGTGTACGTTGG |  |
| **1043** | YFV-17D(-)7278 Reverse | TGCTGCGCTTTGATTCCAGGTA |  |
| **1044** | YFV-17D(+)6900 Forward | TGCTGGAGAAAACCAAAGAGGA | E |
| 156 | YFV-17D(-)7500 Reverse | CCTAAGGCAGCTGATGCTAGG |  |
| 331 | YFV-17D(+)7650 Forward | GAAAAACTTTGGGTGAAG |  |
| 332 | YFV-17D(+)8100 Forward | TCACAGAGGGGGAAAGG |  |
| 333 | YFV-17D(+)8750 Forward | AGGAAGATCATGAAAGTTG |  |
| **1045** | YFV-17D(-)9100 Reverse | CTCAAACTCAAGATACCGCGCT |  |
| **1046** | YFV-17D(+)8749 Forward | TAGGAAGATCATGAAAGTTGTCAACAGG | F |
| 54 | YFV-17D(+)9353 Forward | ACATACAAGAACAAAGTGG |  |
| 1123 | YFV-17D(+)9900 Forward | CAGCTTGCCTCAGCAAAGCCTATG |  |
| 76 | YFV-17D(+)10323 Forward | ATGCTGACCTGCAACTGGG |  |
| 853 | YFV-17D(-)10566 Reverse | GGCAAAACTCGTGTGGGG |  |
| **1047** | YFV-17D (-)10862 Reverse | AGTGGTTTTGTGTTTGTCATCCAA |  |

Primer numbers in **bold** were used to generate six amplicons herein referred to as fragments A – F

**Table S3 Primers and probes used for qRT-PCR**

| **Primer/Probe** | **Sequence (5’ - 3’) ^(a)^** | **Source/Target ^(b)^** | **Size** | **Position** |
| --- | --- | --- | --- | --- |
| YFV Forward | TGGCATATTCCAGTCAACCTTCT | YFV-17D  NS3 | 143 | 4645-4667 |
| YFV Reverse | GAAGCCCAAGATGGAATCAACT |  |  | 4767-4788 |
| YFV-MGB Probe | FAM–TTCCACACAATGTGGCATG–MGB |  |  | 4712-4739 |

^(a)^ Reporter dye (FAM) and TaqMan MGB (minor groove binder) quencher probes

^(b)^ Target sequence in the YFV-17D genome (GenBank accession no. X03700)

**Table S4 Nucleotide changes observed (per mouse) in Stamaril^®^ derived YFV-17D after infection in AG129 mice**

|  | Position | Ref. base | Variant base | Aa_Change (Polyprotein) | Type of mutation | Effect | Gene | Mutation Frequency (%) |
| --- | --- | --- | --- | --- | --- | --- | --- | --- |
| **Brain 2** | 2686 | T | Y | F896[L,F] | Transition | missense | NS1 | 10 |
|  | 9596 | C | Y | H3160[H,Y] | Transition | missense | NS5 | 10 |
|  | 1804 | T | Y | - | Transition | silent | E | 14 |
|  | 5590 | T | G | - | Transversion | silent | NS3 | 100 |
| **Brain 3** | 5695 | C | T | - | Transition | silent | NS3 | 100 |
|  | 7766 | T | C | - | Transition | silent | NS5 | 100 |
|  | 7771 | C | T | - | Transition | silent | NS5 | 100 |
|  | 9445 | C | T | - | Transition | silent | NS5 | 100 |
| **Brain 4** | 2649 | A | G | E844G | Transition | missense | NS1 | 8 |
|  | 5590 | T | G | - | Transversion | silent | NS3 | 100 |
|  | 5695 | C | T | - | Transition | silent | NS3 | 100 |
|  | 6052 | G | A | - | Transition | silent | NS3 | 8 |
|  | 7766 | T | C | - | Transition | silent | NS5 | 100 |
|  | 8230 | C | Y | - | Transition | silent | NS5 | 8 |
|  | 8780 | C | Y | - | Transition | silent | NS5 | 8 |
|  | 9445 | C | T | - | Transition | silent | NS5 | 100 |
|  | 10602 | C | Y | NA | Transition | NA | 3'UTR | 25 |
| **Brain 9** | 739 | G | R | - | Trasition | silent | PrM | 7,1 |
|  | 1417 | G | A | - | Transition | silent | E | 7,1 |
|  | 1704 | T | Y | V529[A,V] | Transition | missense | E | 7,1 |
|  | 4084 | A | R | I1322[I,M] | Transition | missense | NS2A | 7,1 |
|  | 4426 | G | M | - | Transversion | silent | NS2B | 7,1 |
|  | 5174 | G | A | A1686T | Transition | missense | NS3 | 7,1 |
|  | 8100 | T | C | V2661P | Transition | missense | NS5 | 100 |
|  | 8298 | A | T | Y2727S | Transversion | missense | NS5 | 100 |
|  | 8812 | C | T | P2898L | Transition | missense | NS5 | 100 |
|  | 9368 | G | R | V3084W | Transition | missense | NS5 | 100 |
|  | 9832 | C | T | C3238V | Transition | missense | NS5 | 100 |
| **Brain 10** | 2095 | C | Y | S659L | Transition | missense | E | 6,6 |
|  | 3599 | T | Y | - | Transition | silent | NS2A | 6,6 |
|  | 3985 | T | Y | - | Transition | silent | NS2A | 13,3 |
|  | 5998 | C | Y | - | Transition | silent | NS3 | 6,6 |
|  | 6757 | T | C | - | Transition | silent | NS4B | 100 |
|  | 8003 | A | R | I2629[I,V] | Transition | missense | NS5 | 6,6 |
|  | 8789 | C | Y | H2891[H,Y] | Transition | missense | NS5 | 6,6 |
|  | 9368 | G | R | V3084[M,V] | Transition | missense | NS5 | 100 |
|  | 10418 | C | Y | NA | Transition | NA | 3' UTR | 6,6 |
|  | 10481 | C | Y | NA | Transition | NA | 3' UTR | 46,7 |
| **Brain 11** | 1207 | A | G | - | Trasition | silent | E | 6,6 |
|  | 1309 | C | Y | - | Trasition | silent | E | 13,3 |
|  | 2077 | C | Y | - | Trasition | silent | E | 6,6 |
|  | 2803 | T | C | - | Trasition | silent | NS1 | 26,7 |
|  | 3184 | G | A | - | Trasition | silent | NS1 | 6,6 |
|  | 3763 | A | G | - | Trasition | silent | NS2A | 6,6 |
|  | 5124 | C | T | P1669L | Trasition | missense | NS3 | 86,7 |
|  | 5590 | T | G | - | Tranversion | silent | NS3 | 26,7 |
|  | 5695 | C | T | - | Transition | silent | NS3 | 33,3 |
|  | 7120 | T | C | - | Transition | silent | NS4B | 86,7 |
|  | 7270 | A | R | - | Transition | silent | NS4B | 33,3 |
|  | 7333 | T | W | - | Transversion | silent | NS4B | 86,7 |
|  | 7496 | T | Y | - | Transition | silent | NS4B | 86,7 |
|  | 7766 | T | Y | - | Transition | silent | NS5 | 33,3 |
|  | 8611 | T | Y | - | Transition | silent | NS5 | 6,6 |
|  | 9368 | G | R | V3084[M,V] | Transition | missense | NS5 | 100 |
|  | 9685 | C | T | - | Transition | silent | NS5 | 33,3 |
|  | 10120 | G | R | M3334[I,M] | Transition | missense | NS5 | 13,3 |

**Table S5 Nucleotide changes observed (per mouse) in PLLAV-derived YFV-17D after infection in AG129 mice**

|  | Position | Ref. base | Variant base | Aa_Change (Polyprotein) | Type of mutation | Effect | Gene | Mutation Frequency (%) |
| --- | --- | --- | --- | --- | --- | --- | --- | --- |
| Brain 5 | 4708 | G | A | - | Transition | silent | NS3 | 67 |
|  | 7250 | C | Y | L2378[L,F] | Transition | missense | NS4A | 17 |
|  | 10163 | G | A | - | Transition | silent | NS5 | 100 |
| Brain 6 | 7159 | C | Y | - | Transition | silent | NS4A | 20 |
|  | 8957 | A | G | K2947E | Transition | missense | NS5 | 10 |
| Brain 8 | 0 | 0 0 | 0 0 | 0 0 | 0 0 | 0 0 | 0 0 | 0 0 |
| Brain 12 | 39 | A | R | NA | NA | Transition | 5'UTR | 6,7 |
|  | 277 | C | Y | - | silent | Transition | Capsid | 6,7 |
|  | 1993 | T | Y | - | silent | Transition | E | 6,7 |
|  | 2101 | T | Y | - | silent | Transition | E | 6,7 |
|  | 2885 | T | Y | F923[L,F] | missense | Transition | NS1 | 13,3 |
|  | 3574 | G | R | - | silent | Transition | NS2A | 13,3 |
|  | 9583 | A | W | - | silent | Transversion | NS5 | 6,7 |
| Brain 13 | 782 | T | Y | - | silent | Transition | M | 6,7 |
|  | 1786 | C | Y | - | silent | Transition | E | 6,7 |
|  | 2668 | A | R | - | silent | Transition | NS1 | 20 |
|  | 8404 | C | T | - | silent | Transition | NS5 | 100 |
| Brain 14 | 3330 | G | R | G1071[E,G] | missense | Transition | NS1 | 13,3 |
|  | 3872 | C | Y | - | silent | Transition | NS2A | 6,7 |
|  | 4154 | C | Y | - | silent | Transition | NS2A | 6,7 |
|  | 10675 | A | R | NA | NA | Transition | 3'UTR | 13,3 |
| Brain 15 | 3496 | C | T | - | silent | Transition | NS1 | 100 |
|  | 3753 | T | W | I1212[N,I] | missense | Transversion | NS2A | 6,7 |

**Table S6 Comparison of the Stamaril^®^ (lot H5105) consensus sequence with (i) tissue-culture passaged, (ii) plaque-purified and (iii) published YFV-17D genomes**

| **Specimen** | | **Base_Change** | **Position** | **Total_Number of Nucleotide_Variants** |
| --- | --- | --- | --- | --- |
| Stamaril  (P2 BHK)^(a)^ | | 4054N>T | 4054 | 5 |
|  |  | 5362C>T | 5362 |  |
|  |  | 5869t>A | 5869** |  |
|  |  | 6626C>T | 6626** |  |
|  |  | 7496t>Y | 7496 |  |
| Stamaril  (plaque purified consensus) | | 5362C>N | 5362 | 4 |
|  |  | 5869t>A | 5869 |  |
|  |  | 6626C>T | 6626 |  |
|  |  | 7496t>N | 7496 |  |
| 17DD vaccine strain (U17066.1) | | 370C>T | 370 | 36 |
|  |  | 643a>G | 643 |  |
|  |  | 883g>A | 883 |  |
|  |  | 1140t>C | 1140 |  |
|  |  | 1436g>A | 1436 |  |
|  |  | 1437a>G | 1437 |  |
|  |  | 1558C>A | 1558 |  |
|  |  | 1946t>C | 1946 |  |
|  |  | 2110g>A | 2110 |  |
|  |  | 2219a>G | 2219 |  |
|  |  | 2220C>T | 2220 |  |
|  |  | 3599t>C | 3599 |  |
|  |  | 3637C>T | 3637 |  |
|  |  | 4013t>C | 4013 |  |
|  |  | 4054N>C | 4054 |  |
|  |  | 4204C>T | 4204 |  |
|  |  | 4612C>T | 4612 |  |
|  |  | 4873g>T | 4873 |  |
|  |  | 4942a>G | 4942 |  |
|  |  | 4957C>T | 4957 |  |
|  |  | 4972g>A | 4972 |  |
|  |  | 5115a>G | 5115 |  |
|  |  | 5153g>A | 5153 |  |
|  |  | 5225a>C | 5225 |  |
|  |  | 5362C>A | 5362 |  |
|  |  | 6070C>T | 6070 |  |
|  |  | 6514t>C | 6514 |  |
|  |  | 6625a>C | 6625 |  |
|  |  | 7571a>C | 7571 |  |
|  |  | 7701g>A | 7701 |  |
|  |  | 7975C>T | 7975 |  |
|  |  | 8029t>C | 8029 |  |
|  |  | 8808a>G | 8808 |  |
|  |  | 9397a>G | 9397 |  |
|  |  | 10243g>A | 10243 |  |
|  |  | 10550C>T | 10550 |  |
| 17D-213 vaccine strain  (U17067.1) | 1431a>C | | 1431 | 6 |
|  | 4054N>T | | 4054 |  |
|  | 5362C>T | | 5362 |  |
|  | 6625a>C | | 6625 |  |
|  | 7497t>C | | 7497 |  |
|  | 10243g>A | | 10243 |  |
| 17D-204 ATCC strain  (X03700.1)^(b)^ | 4054N>T | | 4054 | 8 |
|  | 5641g>A | | 5641 |  |
|  | 6529t>C | | 6529 |  |
|  | 6758a>G | | 6758 |  |
|  | 7319a>G | | 7319 |  |
|  | 9605a>G | | 9605 |  |
|  | 10243g>A | | 10243 |  |
|  | 10454a>G | | 10454 |  |
| 17D_204 vaccine strain (Stamaril^®^) (X15062.1) [Depuy *et al.*] | 4054N>T | | 4054 | 4 |
|  | 5641g>A | | 5641 |  |
|  | 10243g>A | | 10243 |  |
|  | 10722g>A | | 10722 |  |
| YF-17D as in PLLAV | 4025g>A | | 4025 | 9 |
|  | 4054N>T | | 4054 |  |
|  | 6529t>C | | 6529 |  |
|  | 6758a>G | | 6758 |  |
|  | 8212C>T | | 8212 |  |
|  | 8563C>G | | 8563 |  |
|  | 8566t>C | | 8566 |  |
|  | 9605a>G | | 9605 |  |
|  | 10243g>A | | 10243 |  |

^(a)^ Base changes absent in the consensus sequence of Stamaril^®^ (Lot 5105) as determined by direct sequencing immediately after reconstitution of the vaccine but present after two passages (P2) on BHK-21J are considered tissue-culture adaptive mutations (**). These base changes were not yet detectable after single passage on BHK-21J cells.

^(b)^ GenBank accession no. X03700 was chosen as YFV-17D reference genome throughout similar as by Depuy *et al.* [Nucleic Acids Res. 1989;17(19):3989]. A comprehensive phylogenetic analysis of YFV-17D vaccines of all lineages, 17D-204, 17DD and 17D-213 has been published by Stock *et al.* (2012) [Ref. 17].

**Table S7 Nucleotide variations in all (n = 20) plaque purified Stamaril clones**

| **Specimen** | **Base_Change^(a)^** | **Position** | **Total_Nucleotide_Variants** |
| --- | --- | --- | --- |
| Stamaril_P0^(b)^ | 0 | 0 | 0 |
| Stamaril_P2_consensus | 5362C>N | 5362 | 4 |
|  | 5869T>A | 58698** |  |
|  | 6626C>T | 6626** |  |
|  | 7496T>N | 7496 |  |
| Clone1^(b)^ | 4054N>T | 4054 | 1 |
| Clone2^(b)^ | 571C>T | 571 | 5 |
|  | 1787A>G | 1787 |  |
|  | 2524C>T | 2524 |  |
|  | 4054N>C | 4054 |  |
|  | 9101G>A | 9101 |  |
| Clone3^(b)^ | 4054N>C | 4054 | 6 |
|  | 5134A>G | 5134 |  |
|  | 7735G>A | 7735 |  |
|  | 8100T>C | 8100 |  |
|  | 9116A>G | 9116 |  |
|  | 9991A>C | 9991 |  |
| Clone4^(b)^ | 4054N>C | 4054 | 2 |
|  | 9832C>T | 9832 |  |
| Clone5 | 2524C>T | 2524 | 2 |
|  | 4054N>C | 4054 |  |
| Clone6^(b)^ | 4054N>C | 4054 | 1 |
| Clone7 | 4054N>C | 4054 | 1 |
| Clone8 | 4054N>T | 4054 | 3 |
|  | 5362C>T | 5362 |  |
|  | 7496T>C | 7496 |  |
| Clone9 | 4054N>T | 4054 | 2 |
|  | 6779C>T | 6779 |  |
| Clone10^(b)^ | 4054N>C | 4054 | 4 |
|  | 8100T>C | 8100 |  |
|  | 9832C>T | 9832 |  |
|  | 10546G>A | 10546 |  |
| Clone11^(b)^ | 4054N>C | 4054 | 2 |
|  | 10546G>A | 10546 |  |
| Clone12 | 4054N>T | 4054 | 1 |
| Clone13 | 4054N>T | 4054 | 2 |
|  | 7681A>G | 7681 |  |
| Clone14 | 4054N>T | 4054 | 1 |
| Clone15^(b)^ | 571C>T | 571 | 5 |
|  | 1433A>G | 1433 |  |
|  | 2356T>C | 2356 |  |
|  | 2524C>T | 2524 |  |
|  | 4054N>C | 4054 |  |
| Clone16^(b)^ | 4054N>T | 4054 | 3 |
|  | 5362C>T | 5362 |  |
|  | 7496T>C | 7496 |  |
| Clone17 | 649G>A | 649 | 5 |
|  | 4054N>T | 4054 |  |
|  | 5362C>T | 5362 |  |
|  | 6893C>T | 6893 |  |
|  | 7496T>C | 7496 |  |
| Clone18 | 406C>T | 406 | 5 |
|  | 2207G>A | 2207 |  |
|  | 4054N>T | 4054 |  |
|  | 5362C>T | 5362 |  |
|  | 7496T>C | 7496 |  |
| Clone19 | 4054N>T | 4054 | 1 |
| Clone20^(b)^ | 4054N>T | 4054 | 4 |
|  | 5362C>T | 5362 |  |
|  | 6893C>T | 6893 |  |
|  | 7496T>C | 7496 |  |

1. compared to Stamaril^®^ (lot H5150) P0 consensus sequence
2. submitted to NCBI-Genbank with consecutive accession numbers MN708488 - MN708497

** − tissue-culture adaptive mutations (*).

**Table S8 Relative sequence variability in Stamaril (Lot 5105) based on 20 plaque purified virus clones**

| Variant Nt Position | Reference^(1)^ | %A | %C | %G | %T |
| --- | --- | --- | --- | --- | --- |
| 406 | C | 0 | 95.4 | 0 | 4.5 |
| 571 | C | 0 | 90.9 | 0 | 9 |
| 649 | G | 4.5 | 0 | 95.4 | 0 |
| 1433 | A | 95.4 | 0 | 4.5 | 0 |
| 1787 | A | 95.4 | 0 | 4.5 | 0 |
| 2207 | G | 4.5 | 0 | 95.4 | 0 |
| 2356 | T | 0 | 4.5 | 0 | 95.4 |
| 2524 | C | 0 | 86.3 | 0 | 13.6 |
| 4054 | N | 9 | 50 | 9 | 59 |
| 5134 | A | 95.4 | 0 | 4.5 | 0 |
| 5362 | C | 4.5 | 77.2 | 4.5 | 27.2 |
| 5869** | T | 4.5 | 0 | 0 | 95.4 |
| 6626** | C | 0 | 95.4 | 0 | 4.5 |
| 6779 | C | 0 | 95.4 | 0 | 4.5 |
| 6893 | C | 0 | 90.9 | 0 | 9 |
| 7496 | T | 4.5 | 27.2 | 4.5 | 77.2 |
| 7681 | A | 95.4 | 0 | 4.5 | 0 |
| 7735 | G | 4.5 | 0 | 95.4 | 0 |
| 8100 | T | 0 | 9 | 0 | 90.9 |
| 9101 | G | 4.5 | 0 | 95.4 | 0 |
| 9116 | A | 95.4 | 0 | 4.5 | 0 |
| 9832 | C | 0 | 90.9 | 0 | 9 |
| 9991 | A | 95.4 | 4.5 | 0 | 0 |
| 10546 | G | 9 | 0 | 90.9 | 0 |

^(1)^ X03700.1

** − tissue-culture adaptive mutations
